# Establishing a Rat Model of Pelvic Organ Prolapse with All Compartment Defects by Persistent Cervical Tension

**DOI:** 10.1007/s00192-024-05734-2

**Published:** 2024-01-24

**Authors:** Siqi Bai, Chenxi Lu, Qingyu Kong, Zhuowei Shen, Rui Li, Zhen Xiao

**Affiliations:** 1 Department of Obstetrics and Gynecology, First Affiliated Hospital of Dalian Medical University, 222 Zhongshan Road, Dalian, China; 2 Department of Physics, Dalian University of Technology, Dalian, China

**Keywords:** Pelvic organ prolapse, Animal model, Stress urinary incontinence, Urinary retention, Constipation

## Abstract

**Introduction and Hypothesis:**

We hypothesized that applying cervical suction and persistent tension can develop a novel and efficient rat model of pelvic organ prolapse.

**Methods:**

Fifteen rats underwent pilot testing to optimize the protocol. Sixteen rats were subjected to pelvic organ prolapse induction by cervical suction and constant traction, while five rats served as controls. The pelvic organ prolapse rats were assessed by a Rat Pelvic Organ Prolapse Quantification system at different time points, and their diet, urine, and stool were monitored for 21 days. The pelvic organ prolapse rats were also evaluated for urinary incontinence, urinary retention, leak point pressure, and vaginal histopathology at 21 days after operation.

**Results:**

This rat model demonstrated pelvic floor prolapse in anatomic level, as well as physiological variations (urine incontinence, urinary retention) and pathological changes (collagen fracture, decreased collagen density).

**Conclusions:**

This is the first establishment of the pelvic organ prolapse rat model with all compartment defects, which provides a valuable tool for elucidating pelvic organ prolapse mechanisms and evaluating potential interventions.

**Supplementary Information:**

The online version contains supplementary material available at 10.1007/s00192-024-05734-2.

## Introduction

Pelvic organ prolapse (POP) is a common condition that affects women of all ages, seriously compromising their quality of life. It has a high prevalence worldwide, ranging from 3.40% to 10.76%, and creates a significant economic burden on society [1–3]. Despite its common prevalence, the pathophysiology of pelvic organ prolapse is poorly understood. Large epidemiologic studies have shown that aging, parity, and aberrant connective tissue [4–6] are major risk factors for the development of pelvic organ prolapse.

Because the occurrence and development of pelvic organ prolapse in humans takes years to decades, it is difficult to conduct prospective studies on this disease. Animal models provide a good solution to the complexity and ethical issues of disease research. Though gene knockout mice, rats, sheep, rabbits, and nonhuman primates are utilized in POP research, among which rodents are the most commonly used animal models for studying pelvic organ prolapse in women. Its outstanding advantage is their ease of handling, short lifespan, and relatively low cost [7]. Anatomically, the gross connective tissue anatomy of the rodent pelvis closely resembles that of humans [8]. Moreover, rodents have predictably short oestrous cycles and gestation periods, which facilitate the study of POP development by reducing time requirements [9]. Current methods for creating POP rodent models mainly include the use of genetically defective mice, ovariectomy in rats, and simulation of vaginal delivery [10]. However, the existing model construction methods are expensive, complex to operate, and require long testing cycles, which make it difficult to grasp the depth of disease research.

In this work, we have developed a novel model construction method by cervical suction and continuous traction of the cervix. Models produced using this method can simulate anatomical and histological prolapse, as well as the dysfunction of all compartment defects in the pelvic floor.

## Material and Methods

### Animals

**Fig. 1:**
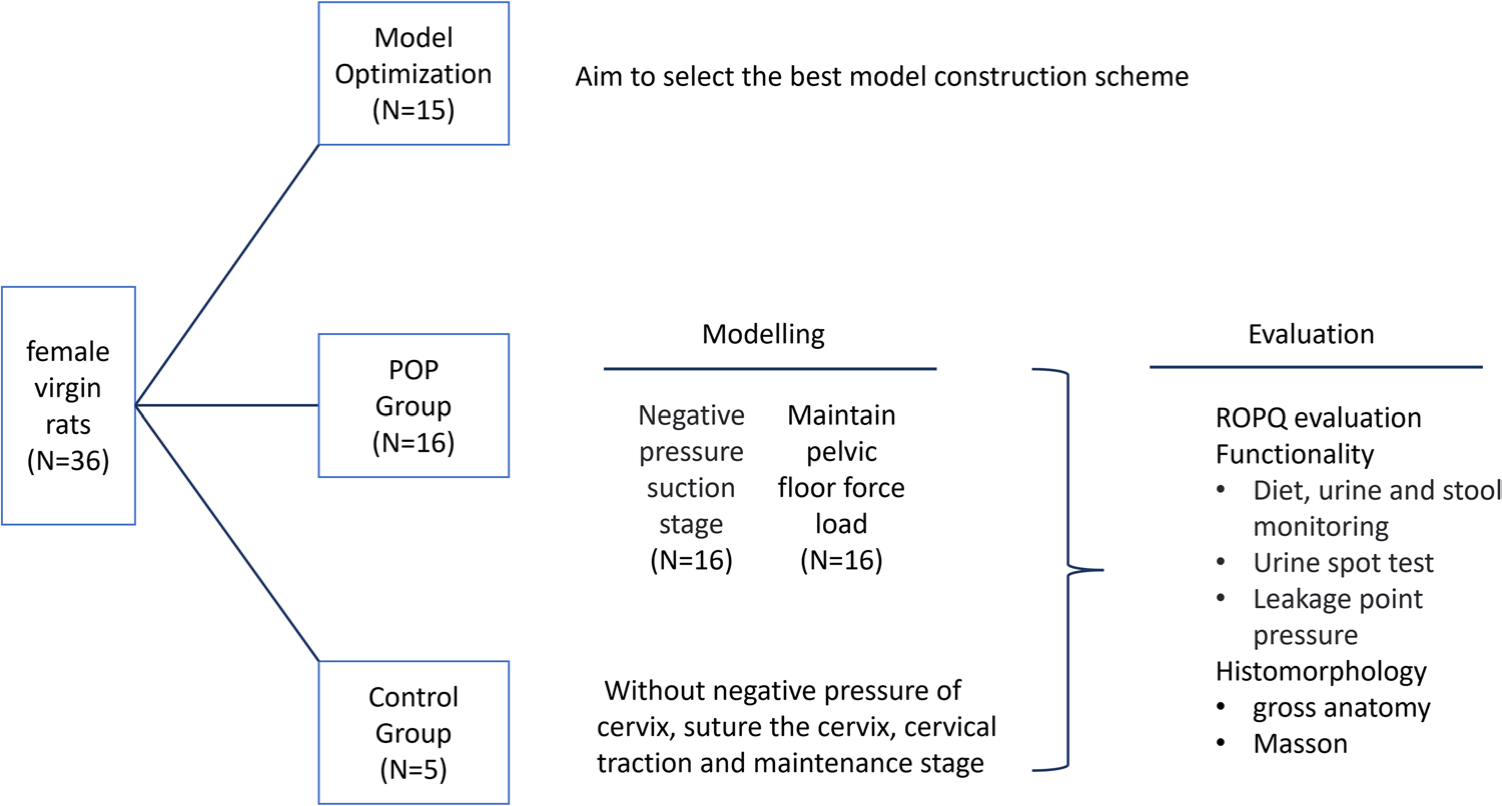
Overview of establishment and evaluation of a POP animal model in rats

This study adhered to the laboratory animal welfare regulations and was approved by the ethics committee on Laboratory Animals of Dalian Medical University (No. AEE23058). We used 36 10-week-old female virgin Wistar rats, with a mean weight of 233.08±10.02 g. The rats were housed in an animal room maintained at a standard humidity (45–50%) and temperature 22±2 °C with 12 h light periods (12 h of daylight/12 h of dark). All animals had access to standard food and water, and were fasted for 12 h before the study procedure, with free water available. The entire experiment was conducted under sterile conditions. The different stages of the experiment are shown in Fig. 1.

### Rat Pelvic Organ Prolapse Quantification System

In order to objectively study the progression of pelvic organ prolapse in an animal model, it is important to use an accurate and reproducible quantification system. Such staging systems have been described in mice [11] and in larger animal models, such as squirrel monkeys and baboons [12, 13]. To our knowledge, there is no description of a staging system for pelvic organ prolapse in rats. Based on the mouse pelvic organ prolapse quantification, we modified a quantification system for rats support to be more suitable for the evaluation of apical prolapse in this study (Table 1). All rats in the POP group were assessed for preoperative prolapse using the ROPQ system before operation. Due to the large range of motion of the rat’s leg joints, it is difficult to ensure that a fixed posture is maintained during each measurement and determine the measurement point of the height of perineal bulge, so it was cancelled. In order to describe apical prolapse, we added distance between cervix and vaginal introitus. Measurements were made in the same position every rat and using callipers with an accuracy of one hundredth of a millimeter (Fig. 3E–G). In addition, we also carried out ROPQ measurements at 3, 7, and 14 days after operation (supplement).

**Table 1:** The ROPQ system

Parameter	Measurement
Grade of perineal bulge	0 = None 1 = Detectable but small 2 = Moderate size bulge 3 = Huge 4 = Vagina coming out
Anal prolapse	0 = None 1 = Present but mild 2 = Severe
Distance between cervix and vaginal introitus^a^	mm
Perineal body length^b^	mm
Genital hernia^c^	mm

^a^Distance between cervix and vaginal introitus: Distance between cervix and vaginal introitus was measured as the distance from the plane of the cervix to the plane of the vaginal outlet in millimeters

^b^Perineal body length: The perineal body was measured in millimeters from the posterior fourchette to the midanus

^c^Genital hernia: The genital hernia was measured in millimeters from the anterior to the posterior vaginal walls at the level of the introitus

### Model Optimization

#### Determination of the Degree of Negative Pressure Suction

**Fig. 2:**
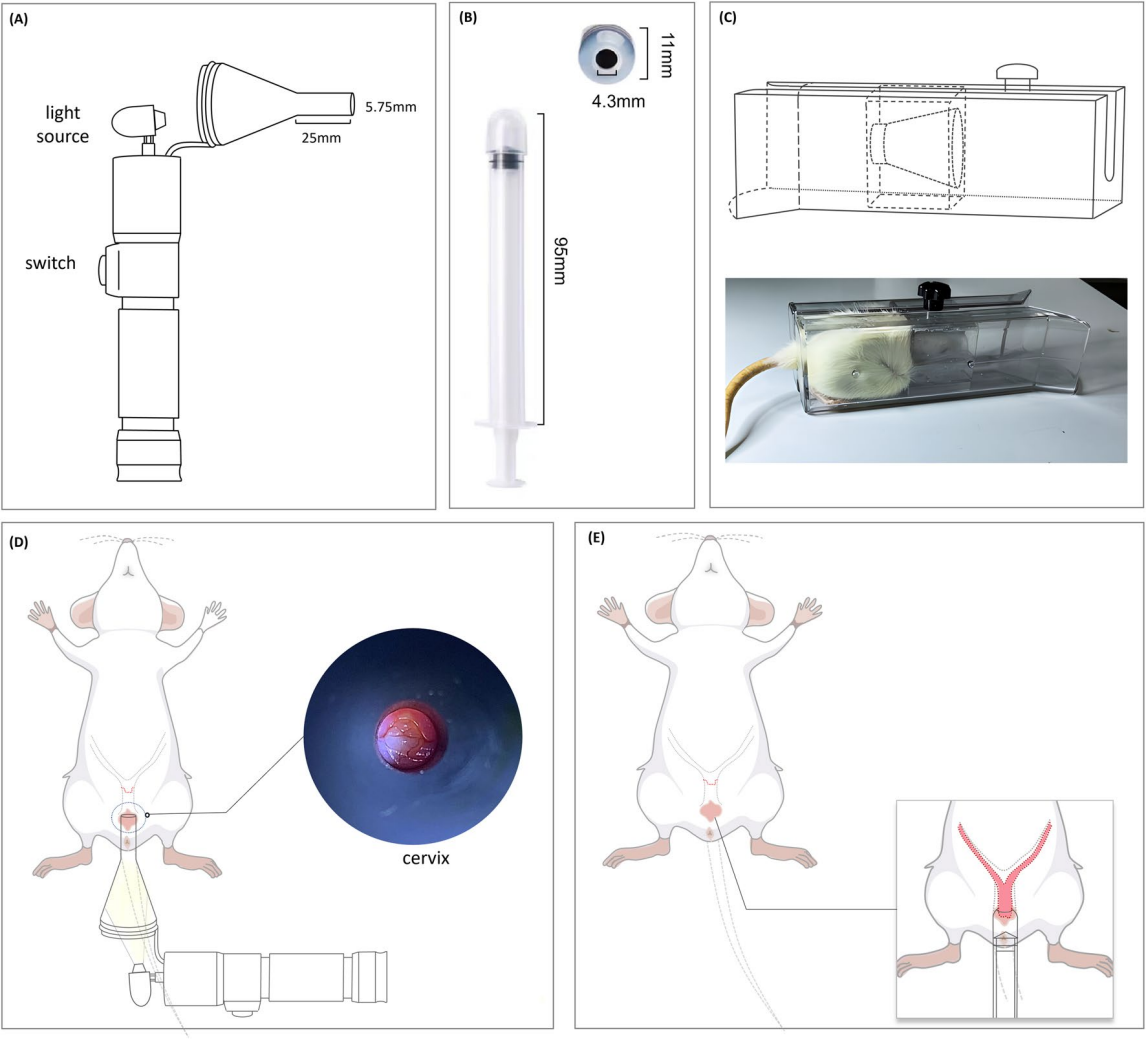
The tool and process examination of cervix, negative pressure suction and fixation device. (**A**) Cervix visualization tool. (**B**) Suction device, the head can accommodate the cervix and a small part of the fornix tissue, and an internal piston with a scale is pulled to provide negative pressure. The negative pressure suction part is 95 mm long, and the inner diameter of the top opening is 43 mm and the outer diameter is 11 mm. (**C**) Fixation device during traction and maintenance phase. (**D**) Schematic representation of rat cervix examined with a cervix visualization tool. The enlarged part on the right is that actual rendering of rat cervix observed by cervix visualization tool. (**E**) Negative pressure suction pattern diagram

The suction device (Fig. 2B) is an opening at the top that can accommodate the cervix and part of the fornix tissue, and the main part is marked with a scale to create a negative pressure state by pulling on the piston. It was placed in the rat vagina with the top opening located below the cervix (Fig. 2E). In the pilot experiment, the downward movement of cervix and uterus was observed under direct vision during negative pressure suction. When the negative pressure suction reaches 1 scale within the device, the position of the cervix and uterus no longer moves down. So we determined the negative pressure attraction as 1 scale in the device. Then slowly move the suction device outward until the cervix is visible at the vaginal introitus and stops. Cross stitch the cervical canal with 3-0 prolene sutures, leaving sutures of 2 cm length for subsequent pulling.

#### Determination of the Tension Frequency and Duration

Fifteen rats were divided into three groups (n = 5 each) and separately modeled them in three protocols that differed in: the frequency of cervical tension per day (5, 7 or 9 times); the duration of each tension cycle (2 min); and the relaxation interval between cycles (1 min). The tension time was determined according to the tolerance and response of the rats to the stretch process. The traction criterion was that the cervix was pulled 5 mm from the external vaginal introitus. The cervixes of rats with nine times cervical tension per day were visible from the introitus on day 7, while those of other groups were visible on day 14 or later. Therefore, the nine times cervical tension protocol was chosen for the formal study.

### Model Construction Process

Under isoflurane anaesthesia, the position and condition of the cervix were observed and detected with the specially made “cervix visualization tool.” There are two main parts, one is the head that a funnel shaped can put into the rat vagina, and the other is the body that with a light source can be held (Fig. 2A). The suction device was placed in the vagina of the rat (Fig. 2D) and negative pressure suction was performed according to the pre-experimental protocol. Finally, the cervix, vagina and anus of the perineum were disinfected, and the rats were placed in a transit cage to recover from anaesthesia.

The POP group was given nine times cervical tension treatment every day within a week after operation. In detail, after the rats were put into fixation device (Fig. 2C). Then given cervical and vaginal surface anaesthesia, the cervical suture was clamped, and the cervix was slowly pulled toward the vaginal introitus until the cervix was pulled out for 5 mm beyond outer vaginal introitus. Each rat was tensioned nine times a day, and each cycle was pulled for 2 min and relaxed for 1 minute. The total duration of the pull and release was 27 min per rat every day, during the rat was always inside the fixation device. The device had the effect of increasing the abdominal pressure of rats, so in the maintenance phase (defined as the rats were placed in the fixation device to increase abdominal pressure, but no cervical suture traction was performed), the rats in the POP group were fixed inside the device for the same time as the traction phase, but no traction was given.

On the 8th day after operation, traction was stopped and the rats entered the maintenance stage, which is the maintain pelvic floor force load phase. The total time in this stage was consistent with the tension stage, that is, each experimental rat maintained the pelvic floor force load for 27 min a day, once a day for one week. The control group rats did not undergo modeling surgery (including negative pressure of cervix, suture the cervix, and cervical traction) and maintenance stage. The results evaluation were the same as the POP group.

### Diet, Urine, and Stool Monitoring

From the first day after operation, the rats were given a fixed amount of food and water at 20 o 'clock every night and were measured at 8 o 'clock the next day for 21 days.

### Urine Spot Test

During the urine spot test, two groups were fed in a polypropylene cage (M-4) of 370*260*170 mm. A 320*225 mm black metal mesh was placed inside, and a 310*225 mm filter paper was placed under the metal mesh (Fig. 5A-B). The metabolic cage was cleaned before the test and dried after alcohol disinfection. After the test began, the water was forbidden for 4h, during which the food was adequate. After 4 h, took out the filter paper, dried it for 24 h, and fixed and colored the urine spots on the filter paper with 0.2% ninhydrin color developing spray.

### Leak Point Pressure Measurement

On day 21, the leak point pressure was measured using the Madlab bioinformatics medical signal acquisition and processing system (MadLab-4C/5H), in order to help to examine whether prolapsed rats developed the condition of urinary incontinence. The rats were anesthetized, fixed, disinfected, and covered. Under aseptic operation, the pelvis was opened and the bladder was exposed. Then we can see the state of bladder filling, utine was drained and calculated. Afterward, the half urine volume of methylene blue solution was put into the empty bladder. Press the bladder until the methylene blue solution emerges from the urethral opening, then stop the pressure, and record the bladder pressure changes throughout the process, with the peak being the maximum leak point pressure.

### Tissue Processing

After the experimental animals were euthanized using the carbon dioxide inhalation method, the pelvic cavity was opened, the bladder and uterus were exposed. Then the pubic symphysis was disarticulated and the urethra and vagina were exposed. Vaginal wall tissues were removed and stored in a -80 °C refrigerator for follow-up histological staining.

### Masson’s Trichrome Staining

The samples were fixed with 10% paraformaldehyde, dehydrated. Transverse sections of 5 μm thickness were prepared. The specific method is carried out according to the protocol of the reagent. Briefly, tissue slices were fixed in Bouin’s solution and subsequently stained with hematoxylin, acid ponceau, and aniline blue solutions. The resulting slices were then observed and photographed using an optical microscope.

### Statistical Analysis

Statistical analyses and graphical representation were performed using GraphPad Prism 9 (GraphPad Software Inc, USA). Data are presented as mean ± standard deviation. A two-tailed Student’s t-test was used to assess differences between the two groups. The repeated measure analysis of variance was used to evaluate the differences about a variable at different times. The experimental group rats at different time points were compared with the control group rats at 2 weeks after sham surgery. Four of the rats died during the experiment due to intestinal obstruction, and the data of dead rats were excluded. Statistical significance was defined as *P* < 0.05.

## Results

### ROPQ Evaluation

**Fig. 3:**
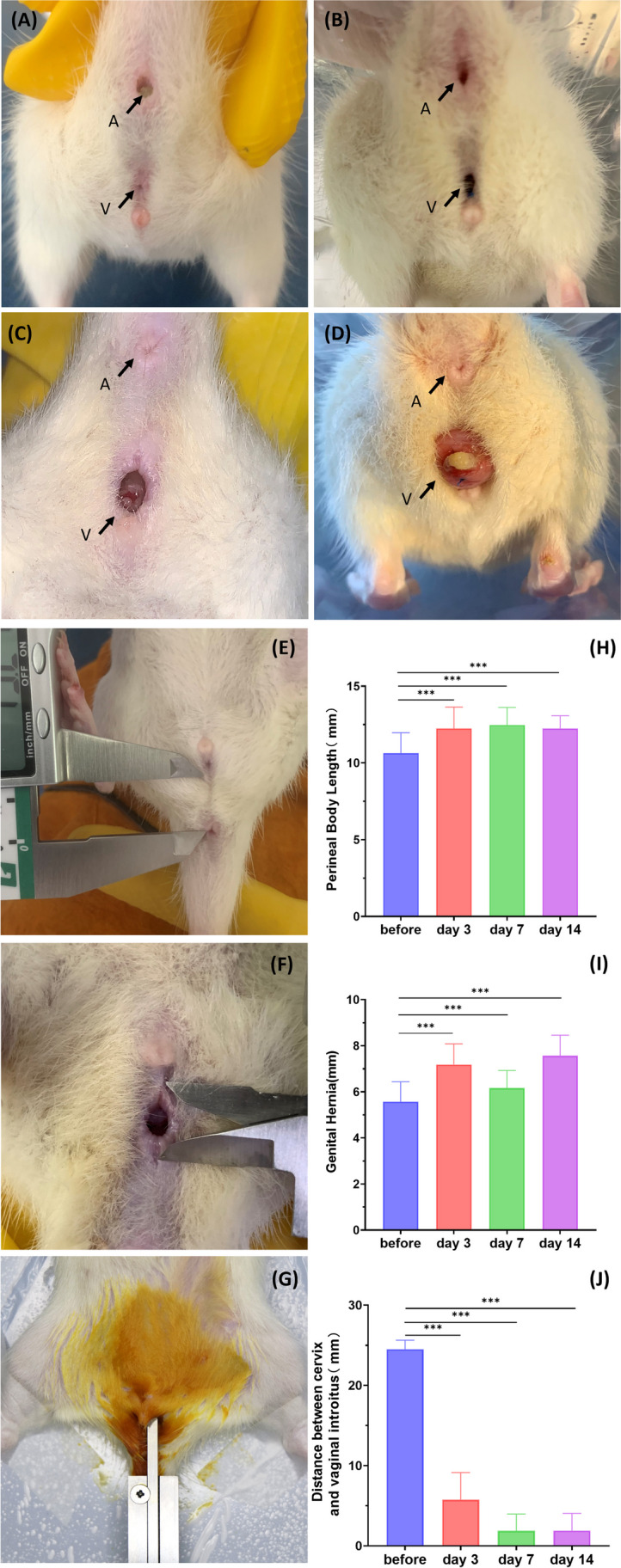
Illustration of the perineal region of the pelvic organ prolapse rats (**A–D**) Before, 3, 7, and 14 days of operation, the rats were affected by regional conditions under Valsalva (A is for the anus, V is for the vaginal introitus). (**E**) Perineal body length. (**F**) Genital hernia. (**G**) Distance between cervix and vaginal introitus. (**H**–**J**) Quantitative statistics of perineal body length, genital hernia, and the length of the cervix from the plane of the vaginal introitus. *, *P* < 0.05. Data are presented as mean ± standard deviation

By observing the prolapse of rats before and 3, 7, and 14 days after operation, the genital hernia underwent a transition from normal to visibly prolapsed tissue under Valsalva (when the rats performed defecation) (Fig. 3A–D). Before operation, the vaginal orifice of rats were nearly closed with no contents protruding. At 3rd day, there was a slight expansion in the genital hernia, but prolapsed contents were not yet visible under direct view. At 7th day, the genital hernia continued to increase and the vaginal orifice can roughly see the contents of the protrusion. At 14th day, the protruded cervix can be clearly seen at the vaginal orifice. The ROPQ system shows that the prolapse of rats was gradually aggravated after operation compared with before, and grade of perineal bulge was almost grade 3 and above on the 14th day. Moreover, grade 3 and above of perineal bulge is accompanied by grade 1 anal prolapse (supplement). In addition, the perineum body length, the genital hernia and the descending distance of the cervix have significantly changed before and after (Fig. 3H–J). Compared with before operation, the perineal body gradually becomes longer, the genital hernia opening becomes larger, and the cervix is gradually closer and closer to the vaginal introitus.

### Leak Point Pressure

**Fig. 4:**
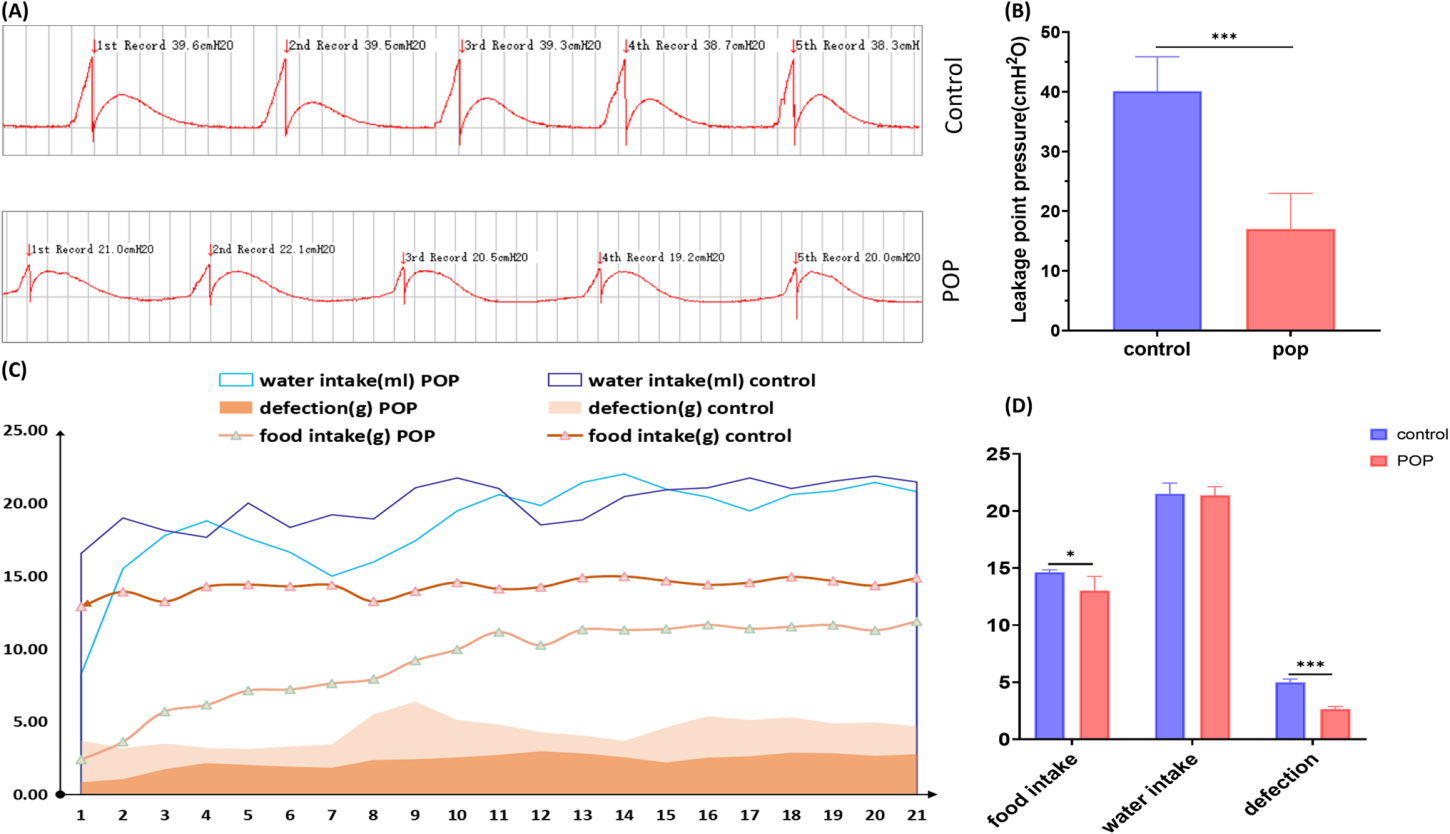
Leak point pressure and diet monitoring (**A**) shows the curve of the test of leak point pressure (peak value is leak point pressure) and the comparison chart of pressure results. (**B**) Results of leakage point pressure analysis. (**C**) shows the records of food intake, water intake, and defection 21 days after operation. (**D**) Statistical analysis results of food intake, water intake, and defection at 14–21 days. *, *P* < 0.05. Data are presented as mean ± standard deviation

During the measurement of bladder pressure, with the increase of external force, the pressure in the bladder gradually increases from the baseline level until it reaches the maximum leak point pressure (Fig. 4A). Then with the withdrawal of external force, the pressure drops instantly, forming the peak position in the curve. The average value was taken after five measurements. Statistics results show the leak point pressure at 21 days after operation was significantly decreased compared with the control group (Fig. 4B).

### Urine Spot Test

**Fig. 5:**
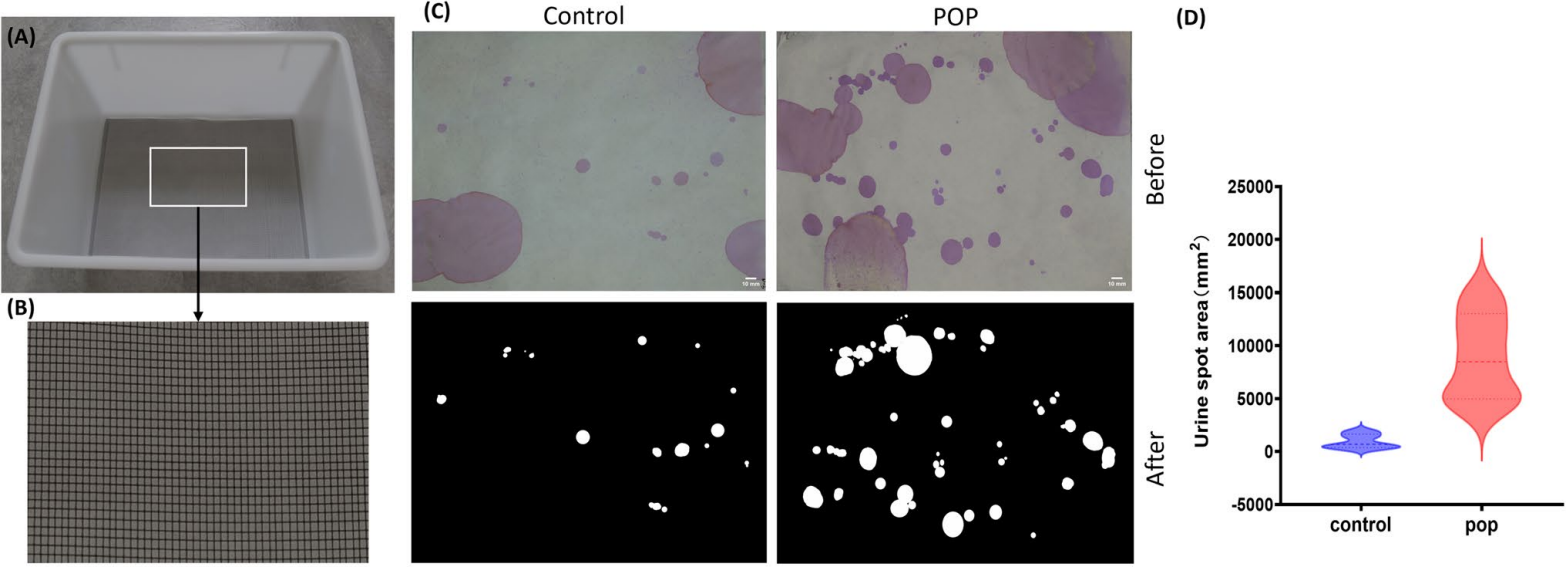
Urine spot test (**A**) The metabolic cage used for the urine spot test. (**B**) shows the wire mesh at the bottom of the cage, with the filter paper positioned below to prevent damage to the filter paper during the experiment. (**C**) The results of the urine spot test of the POP group and the control group. “Before” shows the urine spot on the actual filter paper, and “After” shows the results after removing the large urine pool with image J software. (**D**) The statistical results of the area of urine spots in two groups. *P* < 0.05. Data are presented as mean ± standard deviation

The number and area of urine spots in the POP group at 21 days after operation were significantly higher than those in the control group (Fig. 5C). In addition, statistical analysis of the area of urine spots found that the total area of urine spots in the control group was significantly smaller than that in the POP group, and most of the single area of urine spots in the POP group was also larger than that in the control group (Fig. 5D). This suggests that rats with prolapse could have urinary incontinence.

### Diet and Defecation Monitoring

Water intake, food intake, and defecation of rats after operation were monitored for 21 days (Fig. 4C). Compared with the control group, the food intake of rats in the POP group showed a gradual upward trend within 10 days after operation, which was stable after 2 weeks. However, the food intake was still less than the control group after stability. Especially in the 3 days after operation, the food intake was very small, about 1/6–1/2 of normal food intake. Similarly, the water intake of the POP group fluctuated greatly within 10 days after surgery, and gradually stabilized after 2 weeks. However, the trend of defecation volume in the POP group was almost the same as that of food intake. After 10 days, the volume of defecation was stable, but less than that of the control group. The control group had relatively little fluctuation in water intake, food intake, and defecation. There was a significant difference in food intake and defecation between the two groups, but no significant difference in water intake (Fig. 4D).

### Histopathology Evaluation

**Fig. 6:**
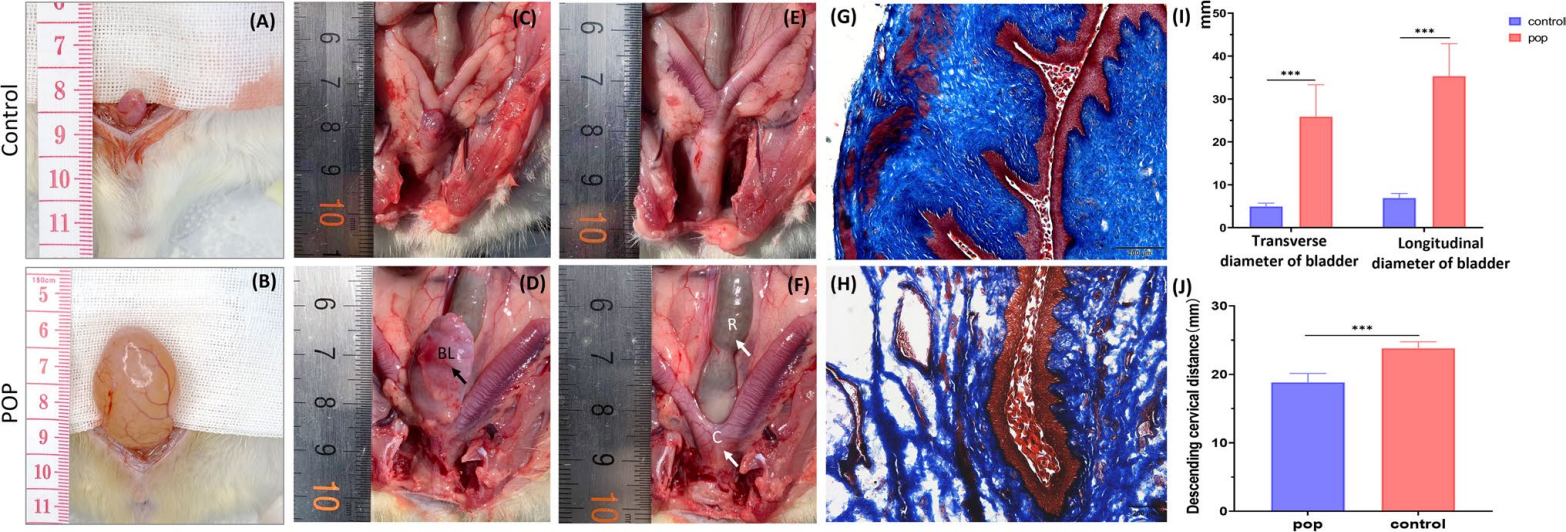
Variations of anatomy and histology in bladder and vagina in POP rat models (**A**, **B**) The comparison diagram of the actual bladder size between the control group and the POP group. (**C**, **D**) The position relationship between bladder and uterus (The BL indicated by the arrow in figure E is the enlarged bladder of the prolapsed rats). (**E**, **F**) The distance between the descending cervical distance in the control and POP group. In panel G, R is the rectum, within which larger fecal masses retained in the rectum can be seen, and C is the descending cervix. (**G**, **H**) Masson staining of the vaginal walls in both groups. (**I**) The statistical result of the bladder size. (**J**) The statistical result of the descending cervical distance. Scale bar = 200 μm. *, *P* < 0.05. Data are presented as mean ± standard deviation

At 21 days after operation, five rats underwent maximum leak point pressure testing in two groups. Then, the rats were humanely euthanized and their pelvic cavities were dissected (Fig. 6). After exposing the bladder by opening the pelvis, it was observed that both the volume and morphology of the POP group’s bladder were significantly greater than those of the control group. In additions, there were significant differences in the two groups in both transverse and longitudinal diameter of bladder (Fig. 6A–B, I). After the pubic symphysis is opened, the bladder and urethral structures become clearly visible, with the urethra situated in close proximity to the anterior wall of the vagina. Moreover, in the control group, the bladder was situated anterior to the angle of bicornuate uterus. However, the bladder had traversed beyond the angle of bicornuate uterus and exhibited an increased volume in the POP group (Fig. 6C–D). Notably, after excision of the bladder and urethra, uterine and vaginal structures are clearly visible (Fig. 6E–F), with a significant reduction in vaginal length. In the POP group, the cervical position was significantly lower than that in the control group (Fig. 6J). Masson staining showed that the lamina propria layer, compared with the control group, the collagen fiber of the vaginal wall was loose. There was an obvious separation among collagen fibers, and the red-stained muscle fibers in POP also showed discontinuous changes (Fig. 6G–H).

## Discussion

At present, establishing animal models is still a challenge in POP-related studies. The primary reason is that current animal models have prolonged model construction periods, high economic costs, and species variations, rendering it challenging for POP animals to fully replicate human prolapse. We have developed a rat model of pelvic organ prolapse by cervical tension and maintaining pelvic floor force load, resulting in typical POP tissue and all compartment defects.

To establish this model, a 3D-printed cervical visualization tool was developed to measure and monitor the cervix size, shape, and position. The cervical suction device enveloped the cervix in a 360-degree manner, resulting in uniform stress distribution and minimal injury. The device simulated the cervical elongation and vaginal expansion induced by the foetal passage through the birth canal, as well as the damage of the supporting structures such as the uterosacral ligament. To induce chronic injury to the connective tissue around the uterus, we maintained cervical tension in the late stage of model creation. As a result of this continuous force, the pelvic organs gradually move away from their original positions, prompting changes in the pelvic floor connective tissue. Because humans are the only strict bipeds, facing a particularly challenging childbirth process and have a unique pelvic orientation in relation to gravity. Therefore, the establishment of relevant animal models for POP remains a formidable challenge. Although the rodent model of POP has some limitations, because the gross connective tissue anatomy of the rodent pelvis is similar to humans, it is still the preferred model for studying the connective tissue of the pelvic floor.

This model simulates the anatomical and histological features typical of POP. Nowadays, diagnosis of POP has depended on clinical diagnosis, such as patient’s symptoms and physical examination [14]. The rat is currently the most applicable animal type for numerous model construction methods [15]. A study comparing the macro- and micro-anatomy of round, uterosacral, and cardinal ligaments in mice and rats concluded that rat pelvic floor structures are histologically more comparable to humans than those of mice [16]. Moreover, many earlier studies on rat model have simulated the morphology of POP at the histological level. For example, Tao Guo et al. constructed a rat POP prolapse model by ovariectomy and simulated delivery and confirmed the presence of prolapse at the histological level [17]. Prolapse at the anatomical level has only been demonstrated in gene knockout mouse models [18] and large animals, such as non-human primate [12, 19], sheep [7, 20], etc. However, our study saw prominent anatomical prolapse in rats by continuous cervical traction on the 7th day (Fig. 3C). Masson staining also confirmed that the collagen fibers of the vaginal wall of the prolapsed rat were significantly sparse and even broken because overdistention of the vagina may cause damage to the fibromuscular tissues, leading to bulging beyond their elastic limits [21], which was extremely similar to the characteristics of human vaginal wall tissue with POP [17, 22].

Concomitant symptoms of prolapse, including mainly urinary incontinence, urinary retention with overflow incontinence and constipation were observed in our models. Symptoms of prolapse in humans, such as incomplete bladder emptying and symptoms of dysfunctional or obstructed voiding, may be indicators of prolapse severity [23]. The previous research has indicated that apical prolapse is responsible for 50% to 60% of anterior wall prolapse, suggesting that the loss of apical support plays a crucial role in the development and/or progression of anterior wall prolapse [24, 25]. Since this method creates a definite apical prolapse, as the apical prolapse increases, which can cause symptoms of anterior and posterior compartment defects. POP and urinary incontinence frequently coexist due to shared etiologic factors in humans [21, 26]. Specific tissue and functional deficiencies resulting in prolapse also significantly contribute to lower urinary tract symptoms, particularly stress urinary incontinence and urinary retention [21]. The urinary spot prolapse rats showed a higher number, total area, and density of spots, indicating stress urinary incontinence. Previous studies have found that anterior vaginal wall prolapse significantly reduces urethral pressure [27]. The prolapsed rats had a significantly lower leak point pressure than the normal rats, indicating that prolapse caused voiding dysfunction. Moreover, POP is also closely related to urinary retention [28]. In this study, the bladder volume of the prolapsed rats was significantly larger than that of the normal rats, suggesting that there may be a manifestation of urinary retention. On the one hand, the cause may be the prolapse itself leading to compression of the bladder neck and urethra. On the other hand, partial pelvic floor nerves could have been injured during cervical suction. Moreover, the dilation of the vagina and the pulling of cervix will also lead to the corresponding mucosal and nerve damage. Although rare, prolapse can cause urinary retention due to compression of the bladder neck and lower urinary tract [29]. Digesu et al. revealed that the severity of the posterior wall prolapse has a stronger relationship with bowel symptoms [30]. It can also be seen from the feeding and defecation that the average food intake and defecation volume of prolapse rats were lower than normal rats during the last week of observation. This may be due to apical prolapse secondary to a posterior compartment defect, resulting in a manifestation of obstructed stool passage, which is the same as constipation caused by posterior wall prolapse in humans.

We were inspired to improve the quantitative system of mice to be more applicable in rats to evaluate the severity of apical prolapse, and named as ROPQ. A previous study has developed a method to quantify the severity of POP in mice, and the results have also reached a certain degree of confirmation and application [11]. Prolapse of vaginal contents can be clearly seen during the Valsalva manoeuvrer, and grade 3 and above prolapse can be achieved in ROPQ (Fig. 3A–D and S1). Moreover, the length of the perineal body was longer than before operation, which may be due to the effect of the digestive system function caused by prolapse. Especially the deposition of feces in the rectum, leading to the expansion of connective tissue around the anus. The diameter of genital hernia was larger than before operation, as the prolapse of vaginal contents makes it difficult to close the vaginal orifice, and may also be related to the expansion of the vagina and the traction of the suture during the operation.

The main limitation of this method is that it does not fully mimic the process of increased abdominal pressure as in humans, so it only applies force to the cervix and para-cervical connective tissue. Despite the presence of anterior and posterior pelvic clinical symptoms, it mainly causes apical prolapse. The use of nulliparous rats in this study did not fully simulate the damage to the pelvic floor caused by childbirth. In addition, due to the short period of time, it is difficult to observe the chronic changes of several years like in human.

Despite some flaws, the model has some strengths. First, the two-step method employed to generate a rat model resulted in significant changes with respect to anatomical features, histopathology and functional levels. Second, our approach established a usable model within 2 weeks compared to other methods, and maintaining stable alterations for up to 3 weeks at least. Third, this method is characterized by reduced time requirements and costs as well as simplicity. Finally, this model does not depend on the animal genetic background. Compared with the vaginal dilatation method, it can better reflect the characteristics of prolapse. It provides a description of bowel and urinary symptoms that is extremely similar to human prolapse, and fills the gap in the prolapse model for post-prolapse manifestations. The animal model established using this method has the potential to be developed into an animal model of POP clinical phenotype.

Given the strengths and limitations, some specific types of studies were potentially suitable for the application of this model. For example, the biocompatibility of exogenous implants, such as artificially synthesized and biological mesh, could potentially be assessed utilizing this model. The model in this study could also be utilized for evaluating the efficacy of stem cell transplantation or immunotherapy. To our knowledge, this is the first study to use the cervical suction method to develop a pelvic organ prolapse. However, our research was only a preliminary exploration of this model. When applied in the future, researchers should specifically verify the corresponding indicators according to their research objectives in order to achieve effectiveness and economy.

### Supplementary Information

ESM 1 (DOCX 673 kb)

## Abbreviations

POP: Pelvic organ prolapse
ROPQ: Rat Pelvic Organ Prolapse Quantification system
