# Supplementary material for: Establishing a Rat Model of Pelvic Organ Prolapse with All Compartment Defects by Persistent Cervical Tension
Source: Int Urogynecol J. 2024 Jan 24;35(3):615–25. doi: 10.1007/s00192-024-05734-2 (PMC11024045; doi:10.1007/s00192-024-05734-2)
Supplement: Supplementary file 1 — (DOCX 673 kb) [file 192_2024_5734_MOESM1_ESM.docx]

**Supplementary picture 1: Grade of prolapse rats.**

Grades 0 (A), 1 (B), 2 (C), 3 (D), and 4 (E). Arrow V indicates area of vaginal bulge. Arrow A indicates area of anal. Grade 0 (A) anal dilation is due to the excretion of feces under the Valsalva, and is not a manifestation of anal prolapse.

**
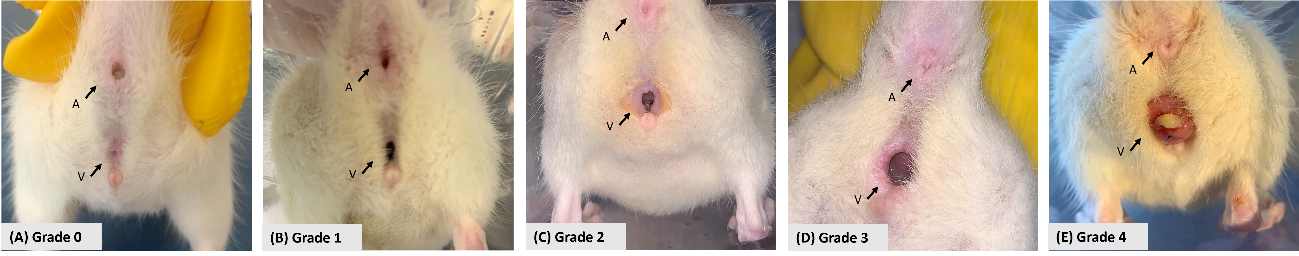
**
